# Quantification of a secondary task-specific tremor in a violinist after a temporal lobectomy

**DOI:** 10.3389/fnhum.2014.00559

**Published:** 2014-07-31

**Authors:** André Lee, Kenta Tominaga, Shinichi Furuya, Fumio Miyazaki, Eckart Altenmüller

**Affiliations:** ^1^Institute for Music Physiology and Musicians’ Medicine, Hannover University for Music, Drama and MediaHannover, Germany; ^2^Department of Engineering Science, Osaka UniversityToyonaka, Osaka, Japan

**Keywords:** tremor, dystonia, epilepsy, task specificity, musicians, coherence, coactivation

## Abstract

Task-specific tremors (TSTs) occur mainly during certain tasks and may be highly disabling. In this case study, we report on a 66-year-old violinist who developed a TST of the right arm only while playing the violin 4 weeks after a temporal lobectomy, which had been performed as a result of his temporal lobe epilepsy. Since a similar case, to our knowledge, has not been reported so far, our aim was to quantitatively assess and describe the tremor by measuring (a) the electromyography (EMG) activity of the wrist flexor and extensor as well as (b) an accelerometer signal of the hand. We found a tremor-related frequency of about 7 Hz. Furthermore, at a similar frequency of about 7 Hz, there was coherence between the tremor acceleration and EMG-activity of the wrist flexor and extensor as well as between the tremor acceleration and coactivation. The tremorgenesis remains unclear, and possible explanations can only be speculative.

## Case study

**Figure 1:**
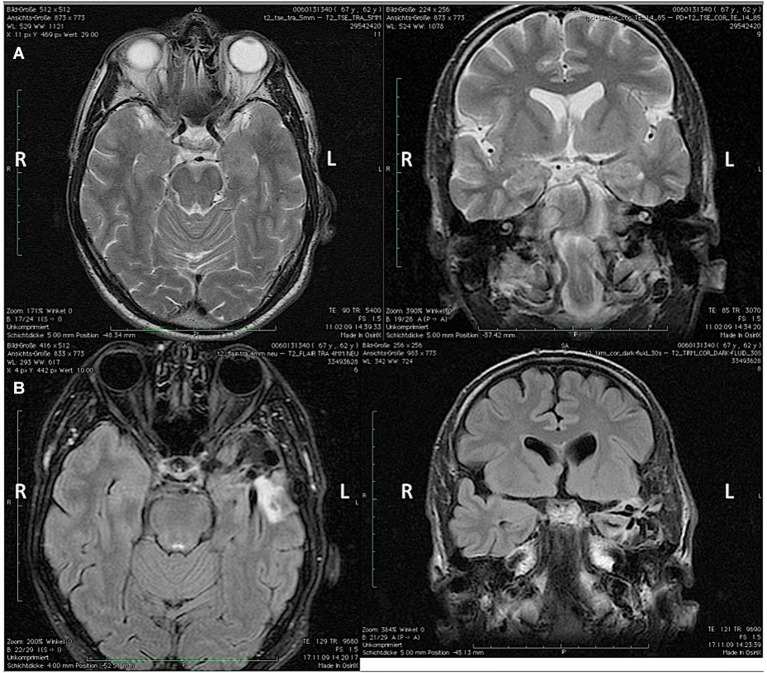
**(A)** Pre-surgery axial (left) and coronal (right) MRI and **(B)** post-surgery coronal (left) and axial (right) MRI showing the apical temporal lobe resection and the shrinkage of the hippocampal remnant. L = Left hemisphere; R = Right hemisphere.

In this paper we report on a 66-year-old, right-handed violinist who came to our institute because of a task-specific tremor (TST) that appeared following a left anterior temporal lobectomy (superior temporal gyrus and corpus amygdaloideum). He had suffered from temporal-lobe epilepsy (TLE) since 2001, and seizures occurred once per month as simple or complex partial seizures. In the 5 years prior to surgery he had had five secondarily generalized tonic-clonic seizures. An interictal EEG revealed intermittent left anterior temporal sharp-waves. Before surgery he had been treated with Lamotrigine 200 mg/day. The pre-surgery MRI showed a slight T2-hyperintensity in the left Amygdala, and the post-surgery MRI revealed a shrinkage of the left hippocampal remnant (Figures 1A,B). A post-resection Electrocorticography showed no epileptic potentials. After the surgery, non-disabling, simple partial seizures occurred (Engel 1B) (Palm Desert International Conference on the Surgical Treatment of the Epilepsies, 1993) as well as a mild anomia that improved over time. The patient was discharged and prescribed with Lamotrigine 200 mg/day. Four weeks following the surgery, he perceived an action-induced, unilateral TST of the right arm that occurred only when he was playing the violin. A follow-up examination included an MRI that showed changes due to a temporal-lobe resection and shrinkage of the hippocampal remnant. An EEG showed no epileptic potentials. The blood count and blood-serum parameters were normal. Upon examination of the patient at our institute, the tremor of the right arm was visible mainly as a flexion-extension tremor of the wrist when slow notes were played on the violin as well as a position-dependent tremor when the arm was held in the position for playing while holding the bow. When the patient did not have the bow in hand, however, he displayed no tremor and there was no rest-tremor. The tremor was not distractible, and there was no entrainment. He was being treated with Mirtazapine 30 mg/d for a reactive depression, which appeared 4 months following the surgery, and Lamotrigine 250 mg/d.

We quantitatively assessed the tremor with regard to its frequency and the coactivation of the wrist flexor and extensor muscle with an accelerometer and a surface-EMG (Biovision, Wehrheim, Germany) while the patient was playing long, slow notes on the open-strings of the violin. We measured the coactivation by calculating the time-varying coactivation of the wrist antagonist muscles and computing the overlap of the waveforms of these muscles (Furuya et al., 2012).

**Figure 2:**
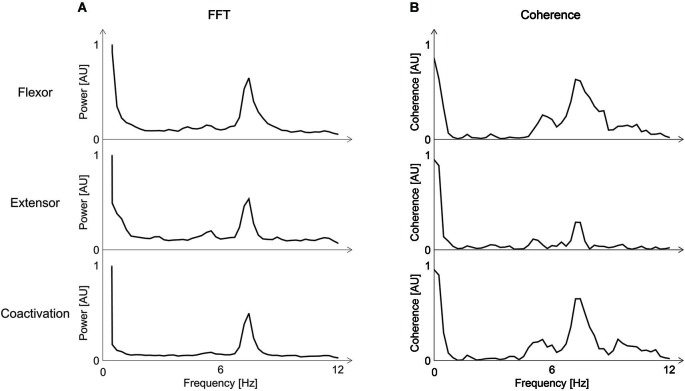
**(A)** Fourier Transform (FFT) of the Electromyogram (EMG)-activity of the wrist flexor and extensor and the coactivation of the right arm. A peak-frequency of about 7 Hz is visible. **(B)** Coherence between EMG-activity for the wrist flexor and extensor and the accelerometer-signal (upper two rows), and coherence between the coactivation and the accelerometer signal (third row). Coherence is highest at the same frequency of about 7 Hz found with the FFT. AU = arbitrary units; FFT = Fourier transform; EMG = Electromyogram.

We then calculated the coherence between the accelerometer data and (a) the EMG-signal; or (b) the coactivation by using the mscohere function in Matlab, specifying 2048-points length of the Hanning window. The coherence calculation provides frequency domain information on the neuromuscular contribution to movement fluctuation (McAuley et al., 1997; Halliday et al., 1999). The results revealed a tremor-related signal with a mean-frequency of 7.5 Hz (Figure 2A) and a coactivation of the wrist flexor and extensor muscles at the same frequency of 7.5 z. Coherence analysis revealed a coherence between EMG-activity and the tremor at 7.5 Hz for each muscle as well as a coherence between the accelerometer and the coactivation of both muscles (Figure 2B). These results are similar to previous results of a study on four patients who suffered from primary bowing tremor (PBT; Lee et al., 2013). In that study, we found a mean frequency of EMG-activity as well as a coactivation for all four patients of 6.6 Hz with a maximum frequency of 7.2 Hz in one patient. In another study assessing PBT, we could show a coherence between the accelerometer signal and the EMG-signals (Lee et al., 2014).

Although tremors can be side effects of Lamotrigine and Mirtazapine, a medication-induced tremor in our patient seemed unlikely to us, because the patient had already taken Lamotrigine before surgery and began taking Mirtazapine only after the tremor had strated occurring. Furthermore, a medication-induced tremor usually does not occur unilaterally or is task-specific. One case-study described a 41-year old woman who developed a psychogenic bilateral kinetic and postural tremor 2 months after having a temporal lobectomy in the upper and lower extremities (Arabi et al., 2012). The tremor disappeared by means of SSRI-medication and psychotherapy (Arabi et al., 2012). Psychogenic disorders after temporal lobectomies have been described before (Glosser et al., 1999; Naga et al., 2004). However, we did not consider the TST of our patient as psychogenic for the following reasons: first, in the majority of cases (Glosser et al., 1999), in fact in up to 90% the cases (Naga et al., 2004) psychogenic symptoms occur after resection in the non-dominant hemisphere. Our patient, though, had undergone surgery in the dominant (left) hemisphere. Furthermore, none of these studies described a psychogenic movement-disorder. Second, we found no feature, such as entrainment or distractibility (Deuschl et al., 1998), which suggested a psychogenic genesis of symptoms.

We cannot exclude the possibility of a coincidence. However, apart from the temporal relation, given that the prevalence of TLE is less than 2/1000 (Hauser and Kurland, 1975 as cited in Téllez-Zenteno and Hernández-Ronquillo, 2012) and that the estimated prevalence of TST is 0.1%, the probability of a coincidence is less than 1:500,000 of a coincidence.

TLE has been described as a network disease with extratemporal effects (Haneef et al., 2014). An involvement of the basal ganglia (BG) in TLE has been described before. In one study a decreased BG-uptake of [^18^F] fluoro-L-dopa was observed in patients with refractory TLE, and this could not be explained by structural changes alone (Bouilleret et al., 2008). Another study could show a projection of the BG onto the temporal lobe which was excitatory (Middleton and Strick, 1996; Gisiger and Boukadoum, 2011) and thought to have a substantial influence on motor areas (Mishkin et al., 1984; Petri and Mishkin, 1994, as cited in Middleton and Strick, 1996). Therefore, in our patient the disruption of this circuit may have led to a deficient excitatory input to the caudate nucleus. This deficient excitatory input to the indirect pathway may have resulted in a reduced inhibitory activity of the Globus pallidus internus (GPi) and thus to a facilitation of movement. A recent study could show in TLE an altered connectivity of the hippocampus to the BG, the cerebellum and to sensory networks including auditory networks. It is known that music making is a highly specialized task requiring a precise temporospatial sensorimotor integration. Training on the instrument usually starts at a young age leading to the plasticity-induced establishment of a multimodal network. This network involves not only in motor areas (Elbert et al., 1998) or the cerebellum (Schlaug, 2001), but also the superior temporal gyrus (Rauschecker et al., 1995; Schlaug, 2001; Bangert et al., 2006) and the hippocampus (Herdener et al., 2010; Papp et al., 2014). Therefore, our case study possibly underlines the importance of a functioning multimodal network in music making, the disruption of which may lead to the loss of fine motor control.

As the pathophysiology of our patient’s tremor remains unclear, our explanation remains speculative, making further investigations necessary.

## Funding

The study was funded by the Institute for Music Physiology and Musicians’ Medicine.

## Conflict of interest statement

The authors declare that the research was conducted in the absence of any commercial or financial relationships that could be construed as a potential conflict of interest.
